# Anxiety in a regular day of work: A 24 hour psychophysiological investigation in young dentists with gender comparison

**DOI:** 10.3389/fpsyg.2023.1045974

**Published:** 2023-02-20

**Authors:** Luca Queirolo, Christian Bacci, Andrea Roccon, Gastone Zanette, Carla Mucignat

**Affiliations:** ^1^Section of Clinical Dentistry, Department of Neurosciences, University of Padua, Padua, Italy; ^2^Department of Philosophy, Sociology, Education and Applied Psychology, University of Padua, Padua, Italy; ^3^Department of Molecular Medicine, University of Padua, Padua, Italy

**Keywords:** dentistry, anxiety, stress, gender, psychophysiology

## Abstract

**Introduction and aim:**

Dentistry is a highly demanding profession with a strong mental and physical involvement, possibly generating anxiety. Very few studies assessed psychophysiological activity in dentists, while none tried to relate it with gender during a routine working day. This study aims at evaluating correlations between gender, psychophysiological indexes, and psychological variables.

**Materials and methods:**

Data were acquired at the Dental Clinic of the University of Padua on 20 healthy young dentists (10 M-10F) during a 24 h period of a working day. Physiological variables (measured with E4 Empatica) were electrodermal activity (EDA), heart rate variability (HRV) and heart rate (HR). Participants anxiety was measured through a self-reported scale on patient-relationship anxiety and through the Generalized Anxiety Disorder-7 Questionnaire (GAD-7).

**Results:**

5 (3F, 2 M) participants over 20 had a GAD-7 score ≥ 10. Female gender, in comparison to Male, was associated with higher perceived patient relationship anxiety (*p* = 0.002) and lower HRV (*p*-adj = 0.022). The gender Male, although being associated with lower level of self-reported anxiety (*p* = 0.002), showed an equal number of subjects with a GAD-7 score ≥ 10 (*p* = 0.371). No interaction between gender and EDA was found, nor an effect of GAD score on EDA, HRV and HR values. Higher values of EDA were found during sleep time; a difference between sleep time and working time EDA (*p* = 0.037) and a difference between sleep time and daytime (*p* = 0.0045). A different HR between sleep and all daytime (*p* < 0.001) was also highlighted.

**Conclusion:**

25% of dentists fell within generalized anxiety disorder diagnosis, compared to a maximum of 8.6% in the general population. A possible general biomarker of excessive stress response was measured: a shift of circadian sympathetic activity was found in dentists; a higher activity during sleep in comparison to working time and daytime. The Female gender was associated with higher perceived patient-approach anxiety, lower parasympathetic activity, and a comparable sympathetic activity to the Male gender, thus fostering a possible vulnerability to excessive stress. This study underlines the need to empower the psychological approach to stress and patient-relationship in dentistry.

## Introduction

Patients’ anxiety for dental procedures is a well-established problem, but who cares about the dentist’s point of view (Armfield and Heaton, 2013)? Stress and anxiety were examined among dental and health professionals and from these studies, it appears that the quality of life of dentists is lower than the general population (Myers and Myers, 2004). Probably this is related to the intrinsic nature of the helping professions (Maslach and Pines, 1977; Rada and Johnson-Leong, 2004), but not solely; being a dentist is a high demanding profession with a strong mental and physical involvement, possibly generating stress and anxiety (DiMatteo et al., 1993). Besides the intrinsic issues of a helping profession, dentists have often to face the management of the dental office. Indeed, the relationship with the patient, the scheduling pressure and the management of employees represent the main causes of stress for dentists (Myers and Myers, 2004). According to some authors, this occupational stress can also lead to a higher risk for cardiovascular diseases (Cutright et al., 1977; Cooper et al., 1978, 1988). The need for professionals to access techniques to manage their stress and improve the communication with the patient is also reported (Moore and Brødsgaard, 2001). It remains to be clarified whether gender differences in anxiety and occupational stress exist. The literature reports a predisposition for anxiety in women, although the factors that may cause this vulnerability remain unclear, Kessler et al. found that the prevalence of a generalized anxiety disorder in women (6.6%) is higher than in men (3.6%) (Kessler et al., 1994; Altemus et al., 2014). Conversely, when anxiety is evaluated in dentists during work, this gender difference seems not to be relevant (Altemus et al., 2014; Davidovich et al., 2015; Asif et al., 2022). The data on anxiety and stress in dentists often derive from questionnaires without ever providing objective measures. However, wearable monitoring devices can measure parameters such as electrodermal activity (EDA), heart rate (HR) and heart rate variability (HRV) and thus provide a step toward an objective psychophysiological measurement of anxiety and stress during the working day (Lu et al., 2020; Posada-Quintero and Chon, 2020; Schuurmans et al., 2020). These psychophysiological data are very important. An increase in HR is associated with increased risk of death (Nauman et al., 2011), more specifically higher HR is associated with both cardiovascular mortality and non-cardiovascular mortality (Woodward et al., 2014; Alhalabi et al., 2017). A reduced HRV is associated with increased cardiovascular risk (Weber et al., 2010), lower resiliency (stress tolerance) in the face of stressful situations (Hansen et al., 2009). Noteworthy longer periods of reduced HRV are associated with worrying, with decreased explicit positive affect, and with increased tension (Verkuil et al., 2016). Furthermore HRV it is highly related to a flexible control over behavior (Thayer et al., 2012), that is hugely important in helping professions such dentistry. The well-known flight or fight memorandum still holds to this day (Cannon, 1915). EDA (being a pure sympathetic index), can provide information on the sympathetic activity and the flight or fight response, because an excessive sympathetic activity can thus fostering healthy related issues (Dickerson and Kemeny, 2004; Andela et al., 2015; Resmini et al., 2016; Richardson et al., 2016; Mohd Azmi et al., 2021). Synthesizing the relevance of all the above-mentioned data, this study aims to investigate the relationships between peripheral psychophysiological parameters measured with a wearable monitoring device and working hours, gender, self-reported anxiety while confronting the patient (VAS-anxiety) and questionnaire measured anxiety (GAD-7) in young dentists.

## Materials and methods

### Participants and setting

Psychophysiological measures and psychological data were acquired from 20 healthy young dentists with at least 1 year of employment attending a post-graduate course at the Dental Clinic of the University of Padua (Padua, Italy). The age of the 20 participants ranged from 26 to 36 years (*M* = 28.95, SD = 2.98), 50% were Male. Exclusion criteria were cardiovascular pathologies or psychiatric disorders. Inclusion criteria were being a dentist at the Dental Clinic of the University of Padua, with less than 5 years of clinical experience. Participants enrolled in the present study have signed informed consent prior to participation. The study was approved by the Ethical Committee of Department of General Psychology, University of Padua, *n*. 3274/2019.

### Procedure

Participants were invited and informed about the aim of the study through an information leaflet, and written informed consent was obtained from all subjects after they received all necessary information on the study. Participants were asked to wear a Empatica E4 (Empatica Inc.) wristband, for 24 consecutive hours while they were working, during sleep and during other daytime activities. Furthermore, subjects were asked to fill-in a diary reporting start and end time of the three different conditions (“work,” “sleep,” “daytime”). Data were collected between March and July 2022.

### Anxiety assessment

Anxiety levels were scored with the Generalized Anxiety Disorder questionnaire GAD-7 and with a Visual Analog Scale (VAS) about Self Perceived Anxiety (Spitzer et al., 2006; Swinson, 2006; Facco et al., 2011). Anxiety scores during the clinical activity were obtained through a self-reported VAS, stating a single item question: “on a scale from 0 to 10, were 0 is nothing at all and 10 is extremely, how do you rate yourself anxious while relating to patients”? A value of 7 (or more) at VAS analog test was taken as the cutoff in defining subjects anxious in relating to patients (ANX+). Participants that reported a VAS-anxiety<7 were defined “ANX−”. GAD-7 was also used as gold-standard measure to report general anxiety symptoms. A value of GAD ≥10 was considered as a positive GAD and defined as “GAD+,” while a value <10 was reported as “GAD−”.

### Physiological assessments

The physiological parameters were obtained through Empatica E4, a wearable device in the form of a wristband that allows for measuring Electrodermal Activity (EDA), blood volume pulse – from which heart rate (HR) and heart rate variability (HRV) are derived, skin temperature, and movement (Garbarino et al., 2014). EDA is a property of the skin that underlines the variation of the electrical conduction in response to sweat secretions and it is a sympathetic index (Boucsein, 2012). HRV is the physiological phenomenon of variation in the time interval between heartbeats. It is a parasympathetic index that reflects vagal activity, it is measured with the root mean square of the successive differences between inter beats intervals (Ernst, 2017; Kim et al., 2018). Participants were asked to wear the Empatica E4 for 24 h on their non-dominant hand. HR was expressed in beats per minute (bpm) and derived through Empatica algorithms to the blood volume pulse. They provide also the inter beats intervals (IBI) from photoplethysmography (PPG) signal. HRV was obtained by extraction of the root mean square of successive differences between normal heartbeats (RMSSD) extracted by first calculating from IBI each successive time difference between heartbeats in *ms*, over a short-term period of 30 s. Then, each of the values was squared and averaged before the square root of the total was obtained. The sensor used to detect blood volume pulse is a PPG sensor, which is known to be subject to missing data as a result of movement or pressure artifacts (Chen et al., 2015). Artifacts were removed, discarding zero values and other single data point outliers. The analysis of EDA included the extraction of a parameter called skin conductance level (SCL). The electrodes used were silver coated with copper underlay on brass electrodes. The threshold for the amplitude of significant signal was set to a minimum rise of 0.005 μSiemens. SCL values were then normalized using the min-max method. Physiological data were down-sampled to 1hz and labeled as belonging to one of the three monitoring periods (defined as condition “work,” “sleep” and “daytime”). Data of each subject were then aggregated into periods, expressing their mean values.

### Statistical methods

As a first step, characteristics of subjects with anxiety (*N* = 5; GAD≥10) were compared to subjects with no anxiety (*N* = 15; GAD<10) using independent *t* tests and a chi-square test, the same analysis was performed splitting groups according to Self-Perceived Anxiety cut-off. We then evaluated if the physiological parameters differed among subjects during the three periods of the day (factor “condition,” 3 levels, within-subjects) and according to the anxiety symptoms reporting both using GAD-7 and Self Perceived Anxiety, (factor “anxiety,” 2 levels, between subjects). The statistical model to test this was a two-way mixed ANOVA model. We then repeated the analysis considering gender differences (factor “gender,” 2 levels, between-subjects) and anxiety symptoms reporting. The analyzes were performed with R version 3.5.1, also used to check for assumptions of normally distributed residuals on all levels of the model. Separate models were considered for each type of parameter (EDA, HR, and HRV). Post-hoc tests were then performed using Tukey for multiple comparisons.

## Results

Male and Female groups were comparable (Table 1). An initial qualitative outcome was that 5 participants showed a GAD-7 score ≥ 10 (3F, 2M) and that 10 participants reported a VAS anxiety ≥7 (7F, 3M). Chi-square statistics showed a significant imbalance of Gender distribution as regards Self-Perceived Anxiety [χ^2^(1,30), *p* = 0.002], a higher number of women in comparison to men reported anxiety in confronting the patients (Table 2). However, there was no effect of Gender distribution when considering GAD+ vs. GAD-population (*p* = 0.371; Table 3). Therefore, Two-Way Mixed ANOVA analyzes were performed considering only GAD as anxiety factor.

**Table 1:** Participant characteristics according to gender.

	Female *N* = 10 Mean (SD)	Male *N* = 10 Mean (SD)	value of *p* (*X*^2^/*t*-test)
Age	27.9 (1.91)	29.1 (3.81)	0.390
Height (cm)	169 (4.62)	177 (5.97)	0.004
Weight (kg)	59.4 (14.2)	72.0 (9.36)	0.041
Body mass index	20.8 (4.52)	23.0 (2.29)	0.215
GAD score	7.70 (5.08)	5.40 (4.33)	0.290

The two groups were comparable according to age, weight, BMI and GAD score.

**Table 2:** Gender factor and EDA, HRV, HR values in ANX− and ANX+ dentists.

		ANX − (*N* = 10)	ANX + (*N* = 10)	Total (*N* = 20)	value of *p*
Gender factor					0.002*
	Female	3 (30.0%)	7 (70.0%)	10 (50.0%)	
	Male	7 (70.0%)	3 (30.0%)	10 (50.0%)	
EDA					0.392
	Mean (SD)	0.120 (0.099)	0.101 (0.067)	0.111 (0.085)	
	Range	0.032–0.414	0.017–0.279	0.017–0.414	
HR					0.423
	Mean (SD)	77.262 (11.541)	79.627 (11.144)	78.444 (11.311)	
	Range	54.292–91.993	48.124–96.388	48.124–96.388	
HRV					0.819
	Mean (SD)	64.613 (19.381)	63.469 (19.152)	64.041 (19.112)	
	Range	41.182–130.594	31.951–97.734	31.951–130.594	

Chi-squared statistics showed a significant imbalance of gender distribution concerning self-perceived anxiety [χ^2^ (1,30), *p* = 0.002]. A higher number of women in comparison to men reported perceived anxiety. ANX − = VAS-anxiety < 7; ANX + = VAS-anxiety ≥ 7; * = *p* < 0.05.

**Table 3:** Gender factor and EDA, HRV, HR values in GAD − and GAD + dentists.

		GAD − (*N* = 15)	GAD + (*N* = 5)	Total (*N* = 20)	value of *p*
Gender factor					0.371
	Female	7 (46.7%)	3 (60.0%)	10 (50.0%)	
	Male	8 (53.3%)	2 (40.0%)	10 (50.0%)	
EDA					0.423
	Mean (SD)	0.116 (0.091)	0.095 (0.060)	0.111 (0.085)	
	Range	017–0.414	0.017–0.239	0.017–0.414	
HR					0.316
	Mean (SD)	77.591 (11.867)	81.004 (9.339)	78.444 (11.311)	
	Range	48.124–96.388	59.470–92.643	48.124–96.388	
HRV					0.117
	Mean (SD)	66.279 (20.279)	57.326 (13.506)	64.041 (19.112)	
	Range	36.097–130.594	31.951–82.811	31.951–130.594	

Chi-squared statistics did not show any significant correlation. 40% of Male and 60% of Female subjects scored ≥ 10 according to the GAD−7 questionnaire. GAD − (it means a GAD−7 score < 10); GAD + (it means a GAD−7 score ≥ 10).

### Electrodermal activity

**Figure 1:**
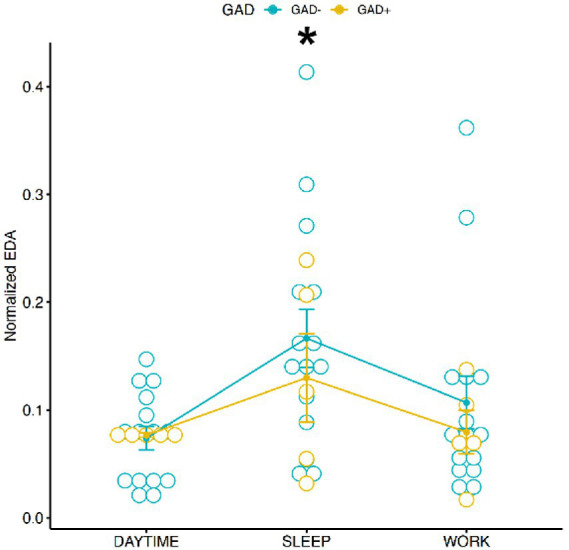
Distribution of normalized EDA during the 3 conditions according to GAD ±. There was no difference between groups according to factor GAD-or GAD+ while EDA values were clearly different during daytime or work in comparison to sleep. Eda values were higher during sleep than daytime and work conditions. * = *p* < 0.05.

**Figure 2:**
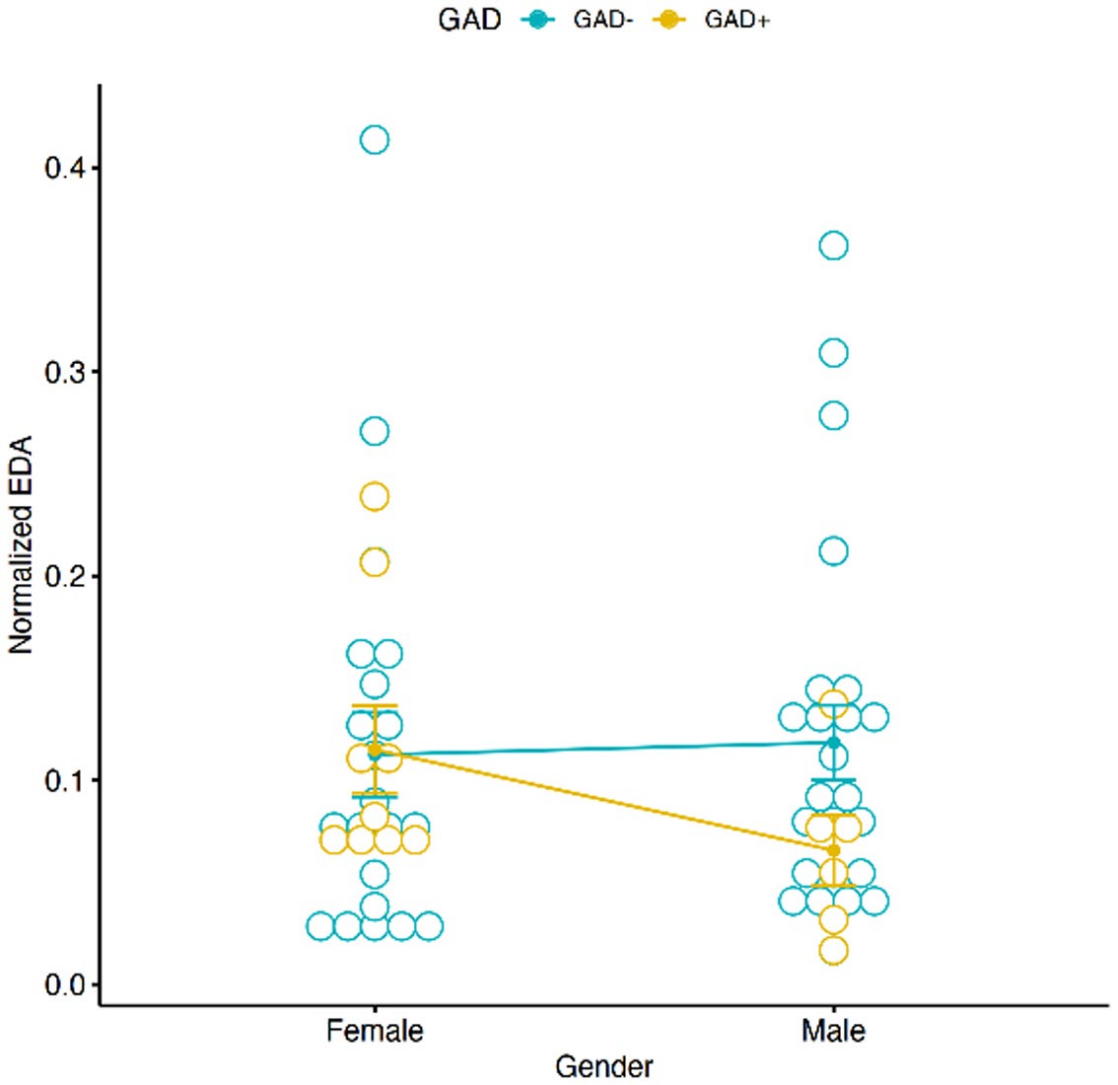
Graphic distribution of normalized EDA according to GAD  ±  and Gender. No significant interaction was found between Gender, EDA and GAD ±.

The results of the Two-Way Mixed ANOVA showed that there was a significant main effect of conditions [*F* (2,54) = 5.2118, *p* = 0.0085] on physiological value EDA. A pairwise comparisons using t-tests with pooled SD revealed significant pairwise differences between conditions “sleep” and “daytime” (*p* = 0.0045) and between “sleep” and “work” (*p* = 0.0367), showing a higher EDA activity during “sleep” in comparison to “daytime” and “work” (Figure 1). There was no interaction effect for EDA, when considering Gender and GAD ± [*F* (1,56) = 1.1432, *p* = 0.2896], with GAD+ (Mean 0.095, SD = 0.091) and GAD− (Mean = 0.116, SD =0.116) performing overall similarly during the different conditions (Figure 2).

### Heart rate variability

**Figure 3:**
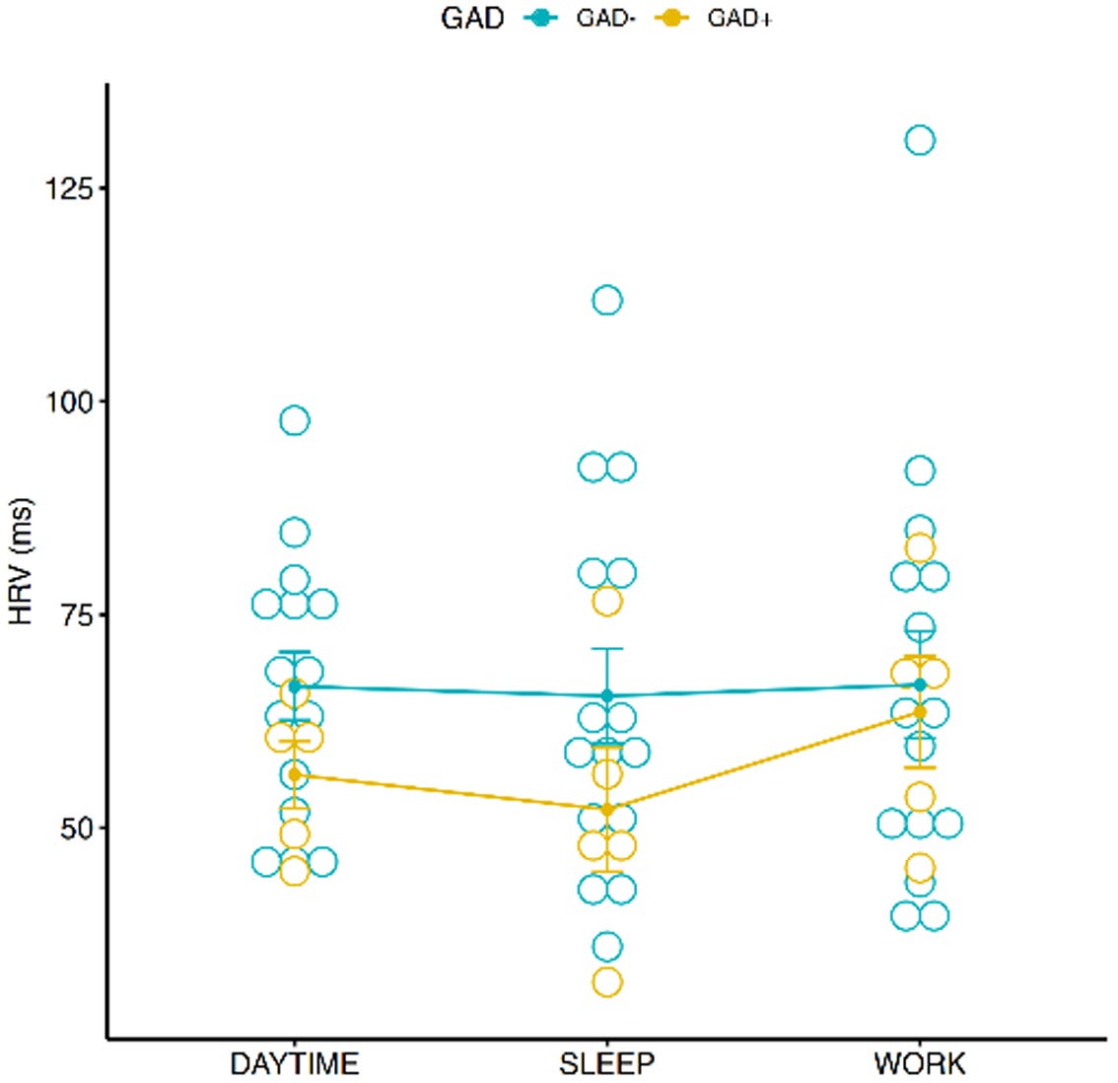
Graphic distribution of HRV values during the 3 conditions according to GAD ±.

**Figure 4:**
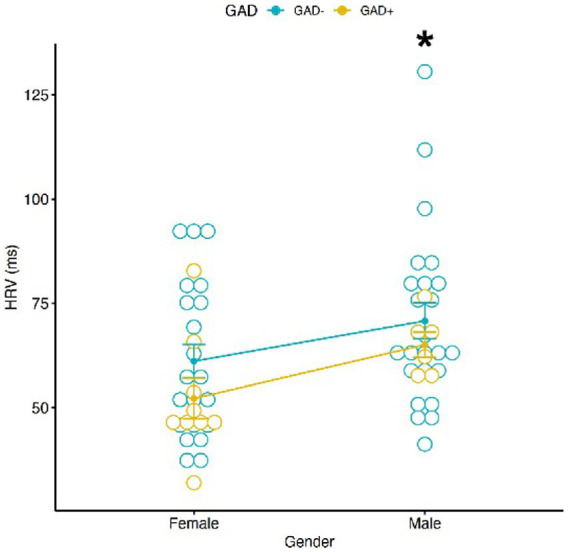
Graphic distribution of HRV values according to GAD ± and Gender. A significant different HRV value according to Gender was found. No significant interaction was found between Gender, HRV and GAD±. * = *p* < 0.05.

Analysis on HRV did not show any significant correlation with GAD ± and conditions (Figure 3), nor among GAD ± and gender effect (Figure 4). However, the Tukey multiple comparisons of means showed a statistically significant difference between Male and Female HRV values (*p*-adj = 0.022), with Females showing a lower HRV in comparison to Males.

### Heart rate

**Figure 5:**
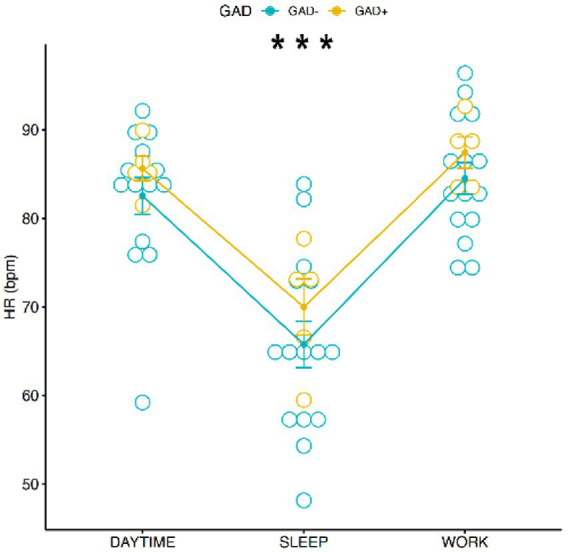
Graphic distribution of HR values during the 3 conditions according to GAD ±. *** = *p* < 0.0001.

**Figure 6:**
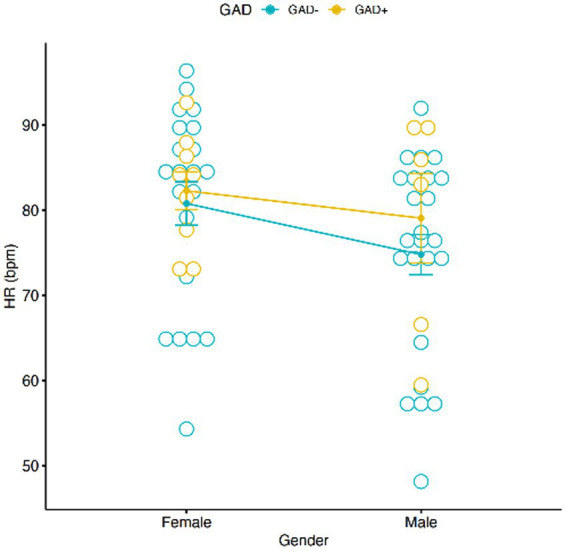
Graphic distribution of HR values according to GAD  ±  and Gender. No significant interaction was found between Gender, HR and GAD ±.

A significant main effect of conditions on HR was found [*F*(2,54) = 25.94, *p* < 0.001; Figure 5], the pairwise comparisons using t-tests with pooled SD showed a significant difference between “sleep” and “daytime” (*p* < 0.001) and “sleep” and “work” (*p* < 0.001). No correlation was found between HR, Gender and GAD ± (Figure 6). Females showed an average HR value 5.59 bpm higher than Males, without reaching the accepted alpha statistical significance level. Tukey multiple comparisons did not show any significative difference between Female and Male HR values (*p*-adj = 0.057).

## Discussion

In this study, several interesting issues have been elucidated. Curiously, neither GAD-7 scores nor self-reported anxiety values interact with the physiological condition in a statically significant way, a result possible due to the limited number of subjects participating. This study nonetheless showed some interesting data from an innovative psychological and physiological perspective. The Female gender is associated with a higher HR (although not statistically significant, but useful, given the consistency of the other parameters, to Figure out a global pattern of activation), higher perceived patient relationship anxiety and, a lower HRV. However, the Male gender although being associated with a lower level of self-reported anxiety, showed an equal number of subjects with a level compatible with a moderate Generalized Anxiety Disorder; a comparable number to the Female group. HR was higher in our Female sample confirming the existing literature, however the difference with the Male sample values was not statistically significant as expected. Maybe this can be attributed to a higher level of anxiety in our Male sample in comparison to the general Male population. Our findings also highlight that women show lower vagal activity compared to men, providing an interesting relationship between self-report anxiety and physiological data. This work underlines differences in HRV values based on RMSSD that are novel compared to the current scientific literature (Koenig and Thayer, 2016). Usually there is no difference between RMSSD according to gender and, if any is reported, Females show higher parasympathetic activity. The current scientific literature has showed the association between an increased morning cortisol concentration and stress levels in dentists (Nijakowski et al., 2022). It has been also highlighted the usefulness of salivary tests to evaluate sympathetic activity (Tammayan et al., 2021). Furthermore in tracking stress the efficacy of oximetry have been already showed (Hunasgi et al., 2018), but no information about dentists’ sympathetic activity during sleep have been previously reported. The present paper highlights differences in EDA circadian rhythms. More specifically dentists maintained a similar sympathetic activity across daytime and working time. In contrast with the literature a higher sympathetic activity was found during the night in comparison to the rest of day (Grassi et al., 2010). This could open some interesting perspectives about EDA values during sleep stages and working day values in order to deepen our understanding of circadian rhythms in dentists that refer to be stressed or actually have anxiety issues. The shift of EDA circadian rhythms (if shown to be consistent by future research) could represent a possible biomarker of work-related stress. Future studies will uncover if in dentists there is any association between nocturnal excessive sympathetic activity, sleep stage, quality of sleep, perceived stress and anxiety. Usually the above-mentioned indexes are associated with bad health and impaired mental health (Sano et al., 2015; Zhang et al., 2018). In the Introduction several important studies were elucidated, that showed the relevance of this psychophysiological perspective, so it will be interesting to evaluate the results of this study not solely according to a Gender comparison but also to the reference population according to age. A comparison with the reference population according to HRV, HR [both defined in Umetani et al., (1998)] and EDA, showed that HRV was higher in our sample 64.041 ms (±19.112) in comparison to 43 ms (±19), reported by the general population paired by age. HR was perfectly in line with the expected results: 79 bpm (±10) in the general population in comparison to 78.444 bpm (±11.311), provided by our sample. EDA values cannot be compared to the general population because a wrist-worn EDA general classification is still lacking, but nonetheless the shift in sympathetic night activity, according to EDA, remains valuable. Noteworthy 50% of participants reported a self-Perceived VAS Anxiety ≥7 and 5 over 20 (3F, 2 M) were compatible with a moderate GAD diagnosis (GAD-7 score ≥ 10). This is more than expected, as reported by Spitzer et al. (2006): the disorder has an estimated current prevalence in the general medical practice of 2.8 to 8.5% and in the general population of 1.6 to 5.0% (Leon et al., 1995; Olfson et al., 1997; Roy-Byrne and Wagner, 2004; Kessler et al., 2005). No interactions between gender and EDA were found, nor an interaction between GAD score and EDA value. It is relevant to underline that probably this lack of interaction may be caused by the limited sample. A further limitation of this study is the impossibility of comparison of self-reported anxiety with gender due to inhomogeneous samples. The relevance of a psychological approach in a very demanding profession such as dentistry was highlighted by the high number of professionals with a moderate generalized anxiety disorder. The Female gender correlation with higher heart rate activity (although non statistically significant), lower parasympathetic activity (HRV) and with comparable sympathetic activity to the Male gender, can thus provide a possible vulnerability to excessive stress. This hypothesis can be confirmed by the higher Female self-reported anxiety related to patient approach. However, this also poses some interrogatives about Males’ perceived stress because GAD-7 Males scores were comparable with Females’ ones and there was no statistically significant difference between Male and Female HR values, giving rise to a question: “Do Females exaggerate perceived anxiety or Males underestimate it?.” Studies with higher numbers are needed to disentangle more clearly the relationship between self-reported anxiety, questionnaires, and psychophysiological activity.

## Data availability statement

The raw data supporting the conclusions of this article will be made available by the authors, without undue reservation.

## Ethics statement

The studies involving human participants were reviewed and approved by Comitato etico della ricerca in psicologia, Dipartimento di Psicologia, Padova. The patients/participants provided their written informed consent to participate in this study.

## Author contributions

All authors listed have made a substantial, direct, and intellectual contribution to the work and approved it for publication.

## Conflict of interest

The authors declare that the research was conducted in the absence of any commercial or financial relationships that could be construed as a potential conflict of interest.

## Publisher’s note

All claims expressed in this article are solely those of the authors and do not necessarily represent those of their affiliated organizations, or those of the publisher, the editors and the reviewers. Any product that may be evaluated in this article, or claim that may be made by its manufacturer, is not guaranteed or endorsed by the publisher.
